# *Mycobacterium avium* ss. *paratuberculosis* Zoonosis – The Hundred Year War – Beyond Crohn’s Disease

**DOI:** 10.3389/fimmu.2015.00096

**Published:** 2015-03-04

**Authors:** Leonardo A. Sechi, Coad Thomas Dow

**Affiliations:** ^1^Department of Biomedical Sciences, University of Sassari, Sassari, Italy; ^2^McPherson Eye Research Institute, University of Wisconsin, Madison, WI, USA; ^3^Chippewa Valley Eye Clinic, Eau Claire, WI, USA

**Keywords:** paratuberculosis, MAP, Crohn’s, autoimmune, molecular mimicry, type 1 diabetes, autoimmune thyroiditis, multiple sclerosis

## Abstract

The factitive role of *Mycobacterium avium* ss. *paratuberculosis* (MAP) in Crohn’s disease has been debated for more than a century. The controversy is due to the fact that Crohn’s disease is so similar to a disease of MAP-infected ruminant animals, Johne’s disease; and, though MAP can be readily detected in the infected ruminants, it is much more difficult to detect in humans. Molecular techniques that can detect MAP in pathologic Crohn’s specimens as well as dedicated specialty labs successful in culturing MAP from Crohn’s patients have provided strong argument for MAP’s role in Crohn’s disease. Perhaps more incriminating for MAP as a zoonotic agent is the increasing number of diseases with which MAP has been related: Blau syndrome, type 1 diabetes, Hashimoto thyroiditis, and multiple sclerosis. In this article, we debate about genetic susceptibility to mycobacterial infection and human exposure to MAP; moreover, it suggests that molecular mimicry between protein epitopes of MAP and human proteins is a likely bridge between infection and these autoimmune disorders.

## Introduction

In 1913, a concise description of what today is known as Crohn’s disease was offered by Scottish surgeon Kennedy Dalziel (1). Twenty years earlier, in 1895, German veterinary Johne H. A. described the cause of an incurable profuse diarrhea in cattle. He noted acid-fast bacteria (most often indicating the organism that causes tuberculosis) that, when transferred to a guinea pig, did not cause tuberculosis (2). Johne first labeled the disease “pseudotuberculosis” and it eventually became known as paratuberculosis.

Infected cow’s intestines had the same cobblestone aspect of Dalziel’s patient and microscopically, the patient’s and cattle’s diseased intestines were so alike that Dalziel wrote that the tissue characteristics were:

… so similar as to justify a proposition that the diseases may be the same (1).

He hypothesized that the disease in cattle and the disease in people shared the same cause. The disease in humans was later named after Dr. Crohn who described a series of patients in 1932 (3).

The heart of this 100-year controversy revolves around the fact that the usual diagnostic techniques to detect bacteria are commonly inefficacious to detect *Mycobacterium avium* ss. *paratuberculosis* (MAP) in humans. A short explanation is that it is just very difficult to grow MAP from humans; and, MAP exists with a modified cell wall – the component of the bacterium that takes up the characteristic acid stain. In this state, the bacterium is no longer “acid fast” and cannot be detected microscopically. Recent work has identified the capacity of MAP to undergo a morphologic change to become spore-like. The spore morphotype survives heat and other stressors and may lead to an increased persistence in hosts and the environment (4).

Understanding the difficulty in detection and appreciating the work of specialty labs that have shown MAP bacteremia in Crohn’s disease patients, there has been a warming to the association of MAP in Crohn’s (5).

## *Mycobacterium avium* ss. *paratuberculosis*

*Mycobacterium avium* ss. *paratuberculosis* is an acid-fast staining small rod-shaped bacterium (6, 7). As with members of the Mycobacteriaceae genus, its cell wall structure rich in complex lipids is unique. The tough and peculiar cell wall of mycobacteria is, in large part, responsible for the persistence of these bacteria, both in the environment and inside the host. Paradoxically, the pathogenic potential of mycobacteria increases as their growth rate decrease. In fact, slow-growing mycobacteria are more pathogenic than fast growing mycobacteria. Except the uncultivable *Mycobacterium leprae* (the cause of leprosy in humans), MAP has the slowest growth rate among harmful mycobacteria. After inoculum of infected samples from infected animals and incubated under optimal conditions, MAP colonies usually appear not before 3 months or more (8).

## MAP and Human Exposure

*Mycobacterium avium* ss. *paratuberculosis* can be found in pasteurized milk (9, 10), milk powder for children (11), surface water (12–14), soil (12), cow manure that contaminates the soil and surface water, moreover cow manure is usually applied as fertilizer in different crops (15) and supply of drinking water (16) all contributing to human exposure. Soil and plants in grazing areas retain MAP; its DNA can be detected in the upper greens of plants, their roots and in the soil below the roots to a depth of 80 cm (17, 18). MAP DNA was detected in over 80% of domestic water samples in Ohio (19). Chlorination and filtration may help to survive mycobacteria rather than eliminate these organisms by killing off their competitors (20). Moreover, mycobacteria organisms have been reported on tap water pipes (21) in biofilms (22) and plastic water bottles (23). One estimate is that mycobacteria could be present in drinking water in “massive numbers,” on the amount of up to 700,000 or 7 × 10^5^ organisms per liter of water (22). A recent study reported testing infant formula for MAP in 65 samples from 18 countries: >40% tested positive for viable MAP (24).

## MAP and Human Diseases

**Figure 1:**
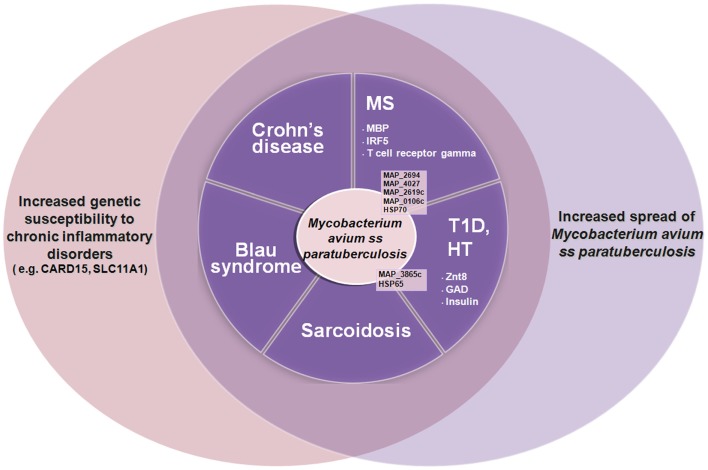
**The increased spread of *Mycobacterium avium* ss. *paratuberculosis* associated with genetic susceptibility to intracellular pathogens such as MAP (e.g., CARD15, SLC11A1) is leading to an increase of autoimmune diseases and inflammatory diseases such as type 1 diabetes (T1D), Hashimoto thyroiditis (HT), multiple sclerosis (MS), Crohn’s disease, Blau syndrome, etc**. Some of the MAP proteins involved are indicated (in black) with the human homologous target proteins (in white).

In addition to Crohn’s, MAP has been associated with multiple diseases: sarcoidosis and Blau syndrome (25), type 1 diabetes (26–32), Hashimoto’s thyroiditis (33–36), and multiple sclerosis (MS) (37–49). In autoimmune diabetes, thyroiditis, and MS, MAP is thought to induce pathology due to molecular mimicry between protein elements of itself and the targeted organ elements of the host, e.g., MAP 3865c and Znt8 in autoimmune (type 1) diabetes and thyroiditis (31, 35, 36). Figure 1 shows how MAP may trigger autoimmune diseases.

If humans are so readily exposed to MAP, why is there not pervasive Crohn’s disease and the other diseases mentioned in this article?

## Genetics

### CARD15

A good example about the interaction between the genetic susceptibility and microbial infection can be found in Crohn’s and Blau syndrome (50), both having polymorphisms within the CARD15 gene.

The gene was originally referred as the NOD2 gene and linkage studies have placed it on chromosome 16; now it is known as the CARD15 gene (51). The CARD15 gene is part of the ancestral innate immune system that recognizes bacteria peptidoglycan in particular mycobacterial glycolylated form of muramyl dipeptide MDP (52–54).

### CARD15, Blau syndrome, and Crohn’s disease

Insights into the consequence of genetic susceptibility to MAP infection may be observed in the rare inflammatory disease, Blau syndrome. This granulomatous inflammatory disorder is characterized by uveitis, arthritis, and dermatitis (50). Although rare, Blau syndrome has been of interest in recent medical literature because of the inherited or *de novo* mutation within the CARD15 gene, the same gene associated with Crohn’s susceptibility (55, 56). However, Blau syndrome susceptibility component of the CARD15 gene is located at the nucleotide binding site domain (55, 56) whereas the Crohn’s susceptibility can be found at the N-terminal leucine-rich repeat domain (57–59).

Blau syndrome shares the same clinical characteristics of juvenile sarcoidosis; in fact, new CARD15 mutations are consistently found in cases of sporadic juvenile sarcoidosis – Blau syndrome (60, 61). For these reasons – the clinical appearance of sarcoidosis and a shared genetic susceptibility with Crohn’s – it was proposed that MAP could have a role in Blau syndrome. A series of Blau tissues comprised of skin, synovial samples as well as Blau graulomas of the liver and kidney were tested for the presence of MAP. Six tissues of five patients representing three different families were all found to have MAP present in the tissue granulomas (25).

The proposed etiopathology is that following MAP exposure, an individual genetically susceptible with mutations within the nucleotide binding domain of CARD15 will exhibit Blau syndrome whereas if the mutations are within the leucine-rich-repeat domain of the same gene, they may exhibit Crohn’s disease. Moreover, it has been reported that CARD15 defects of the leucine-rich-repeat domain, are associated in an aggressive phenotype of Crohn’s disease (62). Recent work has reviewed the susceptibility genes associated with Crohn’s (63).

### SLC11A1

An additional gene linked with Crohn’s susceptibility is the solute carrier 11a1 (SLC11A1) gene (64). SLC11A1 was previously identified as natural resistance-associated macrophage protein 1 (NRAMP1) (65). Polymorphisms within this gene and its promoter are recognized as having a role in the susceptibility of humans and animals to a number of infections, in particular mycobacterial infections, and it has been related to the susceptibility to autoimmune and inflammatory disease as well (64, 65). The SLC11A1 gene, located on chromosome 2q35, is around 14 kb in length. It encodes an integral membrane protein of 550 amino acids that is localized within the acidic endosomal and lysosomal compartment of resting macrophages (65).

The product of the SLC11A1 gene modulates the cellular environment in response to activation by intracellular pathogens by acidifying the phagosome thus killing the pathogen (66). As such, it plays a role in host innate immunity (67). Mutations of SLC11A1 may impair phagosome acidification yielding a permissive environment for the persistence of intracellular bacteria (68).

### SLC11A1 in infectious and autoimmune disease

Sarcoidosis, an other systemic disease associated with MAP, has been associated with polymorphisms of the SLC11A1 gene (69). Susceptibility to mycobacterial diseases, leprosy, and Buruli’s ulcer were also associated with polymorphism of the SLC11A1 gene (70). Similar polymorphisms have been associated with Johne’s disease (paratuberculosis) in cattle (71), goats (72), and sheep (73). A SLC11A1 defect mouse was created by researchers at the Belgium Pasteur Institute to develop a murine model for MAP infection (74).

Due to the capability of SLC11A1 to modulate innate immunity, it is not surprising that the relationship between polymorphisms in SLC11A1 and a number of mycobacterial as well as autoimmune diseases has been explored (75). In addition to leprosy (76) and tuberculosis (77), an association is found in rheumatoid arthritis (78), MS (39, 79), inflammatory bowel disease (80–82), and type 1 diabetes – all diseases associated with MAP (83, 84).

### Molecular mimicry

Molecular mimicry by a microorganisms has been hypothesized to initiate and exacerbate an autoimmune response through sequence or structural similarities with self-antigens (85, 86). Rheumatic fever is one of the best examples for molecular mimicry between group A streptococcus and host antigens leading to the glomerulonephritis and rheumatic heart disease (87, 88). The development of post-streptococcal sequelae is characterized by damage to the heart, joints, and the central nervous system (Sydenham’s chorea). Damage of the heart is the most critical effect and is present in 30–45% of the cases – mostly causing damage to the heart valves.

## MAP and Type 1 Diabetes

Type 1 diabetes mellitus (T1DM) is an autoimmune disease manifest by progressive T cell-mediated autoimmune destruction of insulin-producing beta cells in the pancreatic islets of Langherans (89). Sechi in 2008 found the DNA of MAP in the blood of autoimmune (type 1) patients (32) but not non-autoimmune (type 2) diabetes (27, 28). Sechi also found an association of polymorphisms of the SLC11a1 gene and MAP in T1DM patients (59, 64, 82).

While it may be intuitive to envision an occult presence of MAP as an infective agent producing a granulomatous lesion of Crohn’s or sarcoidosis (Table 1A); it may be more difficult to assign a role for MAP in T1DM. The link connecting MAP and T1DM is molecular mimicry: protein elements of the pathogen “look like” elements of the host’s endocrine pancreas; and immune responses directed at the pathogen sometimes may attack the host (Table 1B). Childhood exposure to cows milk-based infant formula is a strong risk factor for juvenile autoimmune diabetes (30) and, as mentioned in the “exposure to MAP” section of this paper, viable MAP is found in infant formula (24).

**Table 1:** **(A) Map-related granulomatous diseases. (B) Map-associated autoimmune diseases**.

**(A) MAP-RELATED GRANULOMATOUS DISEASES**		
**Disease**	**Shared genetic susceptibility**	**Reference**

Crohn’s	CARD15, SLC11A1	(8, 51, 52, 57, 59, 62, 64)
Sarcoidosis	SLC11A1	(54, 69)
Blau syndrome	CARD15	(52–56, 60)

*These granulomatous diseases are ones where evidence of MAP can be found in the granuloma. CARD15, caspase recruitment domain gene 15; SLC11a1, solute carrier 11a1 gene*.		

**(B) MAP-ASSOCIATED AUTOIMMUNE DISEASES**		
**Disease**	**Mimicking elements**	**Reference**

Autoimmune diabetes	HSP65/GAD	(31, 86–88, 90–94)
	MAP3865c/ZnT8 – pancreatic	
Autoimmune thyroiditis	MAP3865c/ZnT8 – thyroid	(35, 36)
Multiple sclerosis	HSP70, MAP_2694, MAP4027, MAP_2619c 352-61, MAP_0106c protein 121–132	(37–49)

*These autoimmune diseases have autoantibodies. There are share molecular elements between MAP proteins and host organs. HSP65, heat shock protein 65; GAD, glutamic acid decarboxylase; MAP3865c, *M. paratuberculosis* protein 3865c; ZnT8, zinc transporter 8; HSP70, heat shock protein 70; MAP-0106c, *M. paratuberculosis* 0106c protein (aa. 121–132); MBP85-98, myelin basic protein (aa. 85–98)*.

The proposed links is the mimicry of mycobacterial protein MAP3865c and the human homolog Znt8 (31, 35) along with the heat shock protein of MAP (HSP65) and pancreatic glutamic acid decarboxylase (GAD) (30). Different islet autoantibodies (aAbs) may characterize the period preceding T1D clinical onset, aAbs against islets antigens such as insulin, glutamic acid decarboxylase (GAD65), insulinoma associated protein-2, and zinc transporter 8 (ZnT8) may be detectable for months up to years before disease onset. Sechi et al., for example, reported that anti-MAP and anti-ZnT8 antibodies (Abs) targeting homologous membrane-spanning sequences are cross-reactive and capable of eliciting strong immune responses in T1D adult patients (91). One of the sequences was also able to elicit a T cell response (95). An association between MAP and T1D in children was demonstrated by Cossu et al. (96), Additional evidence of the involvement of MAP in the early phases at T1D onset appear from two studies (91, 92) where an association between Abs positive for ZnT8 and MAP homolog epitopes in Sardinian and Italian children at T1D onset was demonstrated. Moreover, Sechi et al. (93) reported a similar high antibody response against insulin epitopes and its MAP homologous peptides in children; those both at risk for T1D and at T1D onset. A review on the topic was previously reported (94).

## MAP and Autoimmune Thyroiditis

The most common autoimmune disease associated to T1D is autoimmune thyroid disease, its frequency is estimated at >90% among patients with T1D and autoimmune diseases (97). Different articles associate MAP to autoimmune (Hashimoto’s) thyroiditis (HT) (33, 34). The same molecular mimicry principle is suggested as the link between MAP and Znt8, one of the organ-specific autoantigens of thyroiditis (33–36). Though ZnT8 is primarily expressed in pancreatic islet cells, it is also expressed in the follicular and para-follicular epithelial cells of the thyroid gland. In view of the evidence accounting for a cross-recognition of MAP3865c/ZnT8 homologs sequences in T1D subjects, and applying the theory which proposes MAP as an HT environmental trigger (acting trough a molecular mimicry mechanism) (35, 36), it is natural to consider MAP for a causal role in HT. Moreover, it has been reported that the occurrence of islet aAbs (especially Znt8) was associated with a positive titer of thyroid peroxidase antibodies (ATPO) in newly diagnosed adult-onset autoimmune diabetic patients (98).

## Heat Shock Proteins

Heat shock proteins (HSPs) are expressed at high level in response to environmental stress. They stabilize proteins and are involved in the folding of denatured proteins helping cells survive stressful conditions and promoting recovery (99). HSPs are synthesized to respond to the presence of invading pathogens. However, pathogens may also produce their HSPs. The increased expression of both self and infective stress proteins and the extensive sequence homology between microbial and human HSP (50–80% amino acid homology of mycobacterial HSP65 and human HSP60) have led to the concept that HSPs are involved in the etiology and pathogenesis of many immune-mediated disorders (100). Antibodies to mycobacterial HSPs have been found in various autoimmune diseases (101). Just to mention some, the mycobacterial 65 kDa HSP has been associated to rheumatoid arthritis (102–104), autoimmune hepatitis (105), primary biliary cirrhosis (106), and systemic sclerosis (107). HSP65 has been reported in different vasculitis-associated systemic autoimmune diseases such as Kawasaki disease (108), Behcet’s disease (109) Takayasu’s arteritis (110), moreover, Hsp70 has also been associated with MS (90).

## MAP and Multiple Sclerosis

Sechi et al. have published studies implicating MAP in MS (37–39, 41–49). Molecular mimicry and SLC11A1 associations are central to this association as well (40, 41). MAP has been associated with Epstein–Barr virus (EBV – thought to be one of the triggers of MS) (44): peptides of each microorganism (MAP and EBV) cross react with anti-myelin basic protein (MBP) (43) and interferon regulatory factor 5 (IRF5) in MS patients (48). Interferon-beta therapy influence antibody response against MAP (49). An extensive review on the topic has been previously published (46).

## The Future – MAP and Human Disease

The role of MAP in Crohn’s disease has progressed from controversial to conspicuous to compelling. The century-old striking similarities existing between Johne’s and Crohn’s diseases on a tissue level are now validated at cellular and molecular levels (90). There is an increasing awareness and call for resolution (111, 112). Improved testing strategies for ruminant herds such as metabolomic profiling (113) will aid in the public health approach to animal disease and sources of human exposure. On a limited basis, Crohn’s disease has been treated successfully with antibiotics (114, 115). As the MAP/Crohn’s debate resolves and as more diseases are linked to MAP, there will likely be a major shift in the public health approach to MAP and human disease. Early indications of such a shift are two clinical trials employing anti-mycobaterial drugs: clarithromycin, rifabutin, and clofazimine. One is a 60-center trial in Crohn’s disease (116) and another is the same treatment for MS (117). Positive outcomes from efforts like these – curing Crohn’s disease and MS with anti-mycobacterial medication as well as prevent autoimmune diabetes and thyroiditis – will further solidify the role of MAP as a zoonotic agent in human disease and, perhaps after more than a century, will resolve this medical controversy.

## Conflict of Interest Statement

The authors declare that the research was conducted in the absence of any commercial or financial relationships that could be construed as a potential conflict of interest.

## References

[1] Dalziel TK Chronic interstitial enteritis. Br J Med (1913) 2:2756.

[2] From history of Johne’s disease. Johne’s Information Center. (2014). Available from: http://www.johnes.org/history/index.html

[3] Crohn BB Ginzburg L Oppenheimer GD Regional ileitis – a pathologic and clinical entity. JAMA (1932) 99(16):1323–9 10.1001/jama.1932.02740680019005

[4] Lamont EA Bannantine JP Armién A Ariyakumar DS Sreevatsan S. Identification and characterization of a spore-like morphotype in chronically starved *Mycobacterium avium* subsp. paratuberculosis cultures. PLoS One (2012) 7(1):e30648. 10.1371/journal.pone.0030648 22292005 PMC3265505

[5] Agrawal G Borody TJ Chamberlin W ‘Global warming’ to *Mycobacterium avium* subspecies paratuberculosis. Future Microbiol (2014) 9(7):829–32 10.2217/fmb.14.52 25156371

[6] Bhamidi S Scherman MS Jones V Crick DC Belisle JT Brennan PJ Detailed structural and quantitative analysis reveals the spatial organization of the cell walls of in vivo grown *Mycobacterium leprae* and in vitro grown *Mycobacterium tuberculosis*. J Biol Chem (2011) 286(26):23168–77. 10.1074/jbc.M110.210534 21555513 PMC3123084

[7] Niederweis M Danilchanka O Huff J Hoffmann C Engelhardt H. Mycobacterial outer membranes: in search of proteins. Trends Microbiol (2010) 18(3):109–16. 10.1016/j.tim.2009.12.005 20060722 PMC2931330

[8] Collins MT. Paratuberculosis: review of present knowledge. Acta Vet Scand (2003) 44:217–21. 15074635

[9] Millar D Ford J Sanderson J Withey S Tizard M Doran T IS900 PCR to detect *Mycobacterium* paratuberculosis in retail supplies of whole pasteurized‘cows’ milk in England and Wales. Appl Environ Microbiol (1996) 62:3446–52. 8795236 10.1128/aem.62.9.3446-3452.1996 PMC168142

[10] Ellingson JL Anderson JL Koziczkowski JJ Radcliff RP Sloan SJ Allen SE Detection of viable *Mycobacterium avium* subsp. paratuberculosis in retail pasteurized whole milk by two culture methods and PCR. J Food Prot (2005) 68(5):966–72. 15895728 10.4315/0362-028x-68.5.966

[11] Hruska K Bartos M Kralik P Pavlik I *Mycobacterium avium* subsp. paratuberculosis in powdered infant milk: paratuberculosis in cattle – the public health problem to be solved. Vet Med Czech (2005) 50(8):327–35.

[12] Pickup RW Rhodes G Arnott S Sidi-Boumedine K Bull TJ Weightman A *Mycobacterium avium* subsp. paratuberculosis in the catchment area and water of the River Taff in South Wales, United Kingdom, and its potential relationship to clustering of Crohn’s disease cases in the city of Cardiff. Appl Environ Microbiol (2005) 71:2130–9. 10.1128/AEM.71.4.2130-2139.2005 15812047 PMC1082532

[13] Whan L Ball HJ Grant IR Rowe MT. Occurrence of *Mycobacterium avium* subsp. paratuberculosis in untreated water in Northern Ireland. Appl Environ Microbiol (2006) 71:7107–12. 10.1128/AEM.71.11.7107-7112.2005 16269747 PMC1287693

[14] Pickup RW Rhodes G Bull TJ Arnott S Sidi-Boumedine K Hurley M *Mycobacterium avium* subsp. paratuberculosis in lake catchments, in river water abstracted for domestic use, and in effluent from domestic sewage treatment works: diverse opportunities for environmental cycling and human exposure. Appl Environ Microbiol (2006) 72:4067–77. 10.1128/AEM.02490-05 16751517 PMC1489623

[15] Grewal SK Rajeev S Sreevatsan S Michel FC Jr. Persistence of *Mycobacterium avium* subsp. paratuberculosis and other zoonotic pathogens during simulated composting, manure packing, and liquid storage of dairy manure. Appl Environ Microbiol (2006) 72:565–74. 10.1128/AEM.72.1.565-574.2006 16391093 PMC1352242

[16] Collins MT Miliotis MD Bier JW International Handbook of Foodborne Pathogens. Boca Raton, FL: CRC Press (2003). 17 p.

[17] Kaevska M Lvoncik S Lamka J Pavlik I Slana I. Spread of *Mycobacterium avium* subsp. paratuberculosis through soil and grass on a Mouflon (*Ovis aries*) pasture. Curr Microbiol (2014) 69(4):495–500. 10.1007/s00284-014-0618-418 24880776

[18] Pribylova R Slana I Kaevska M Lamka J Babak V Jandak J Soil and plant contamination with *Mycobacterium avium* subsp. paratuberculosis after exposure to naturally contaminated mouflon feces. Curr Microbiol (2011) 62(5):1405–10. 10.1007/s00284-011-9875-7 21279514

[19] Beumer A King D Donohue M Mistry J Covert T Pfaller S. Detection of *Mycobacterium avium* subsp. paratuberculosis in drinking water and biofilms by quantitative PCR. Appl Environ Microbiol (2010) 76(21):7367–70. 10.1128/AEM.00730-10 20817803 PMC2976226

[20] Falkinham JOIII. Factors influencing the chlorine susceptibility of *Mycobacterium avium*, *Mycobacterium intracellulare*, and *Mycobacterium* scrofulaceum. Appl Environ Microbiol (2003) 69:5685–9. 10.1128/AEM.69.9.5685-5689.2003 12957962 PMC194915

[21] Falkinham JO III Norton CD LeChevallier MW. Factors influencing numbers of *Mycobacterium avium*, *Mycobacterium intracellulare*, and other mycobacteria in drinking water distribution systems. Appl Environ Microbiol (2001) 67:1225–31. 10.1128/AEM.67.3.1225-1231.2001 11229914 PMC92717

[22] Vaerewijck MJ Huys G Palomino JC Swings J Portaels F. Mycobacteria in drinking water distribution systems: ecology and significance for human health. FEMS Microbiol Rev (2005) 29:911–34. 10.1016/j.femsre.2005.02.001 16219512

[23] Tatchou-Nyamsi-Konig JA Dailloux M Block JC. Survival of *Mycobacterium avium* attached to polyethylene terephtalate (PET) water bottles. J Appl Microbiol (2009) 106:825–32. 10.1111/j.1365-2672.2008.04050.x 19187155

[24] Grant I Foddai A Kunkel B Collins MT Detection of viable *Mycobacterium avium* subsp. paratuberculosis (MAP) in infant formula. Presented at the 12th International Colloquium on Paratuberculosis. Parma (2014).

[25] Dow CT Ellingson JL. Detection of *Mycobacterium avium* ss. Paratuberculosis in Blau syndrome tissues. Autoimmune Dis (2010) 20(2010):127692. 10.4061/2010/127692 21152214 PMC2989750

[26] Cossu A Rosu V Paccagnini D Cossu D Pacifico A Sechi LA. MAP3738c and MptD are specific tags of *Mycobacterium avium* subsp. paratuberculosis infection in type I diabetes mellitus. Clin Immunol (2011) 141(1):49–57. 10.1016/j.clim.2011.05.002 21664191

[27] Rosu V Ahmed N Paccagnini D Pacifico A Zanetti S Sechi LA. *Mycobacterium avium* subspecies paratuberculosis is not associated with type-2 diabetes mellitus. Ann Clin Microbiol Antimicrob (2008) 22(7):9. 10.1186/1476-0711-7-9 18430197 PMC2365959

[28] Rosu V Ahmed N Paccagnini D Gerlach G Fadda G Hasnain SE Specific immunoassays confirm association of *Mycobacterium avium* Subsp. paratuberculosis with type-1 but not type-2 diabetes mellitus. PLoS One (2009) 4(2):e4386. 10.1371/journal.pone.0004386 19204799 PMC2636876

[29] Sechi LA Rosu V Pacifico A Fadda G Ahmed N Zanetti S. Humoral immune responses of type 1 diabetes patients to *Mycobacterium avium* subsp. paratuberculosis lend support to the infectious trigger hypothesis. Clin Vaccine Immunol (2008) 15(2):320–6. 10.1128/CVI.00381-07 18077612 PMC2238046

[30] Dow CT. Paratuberculosis and type I diabetes: is this the trigger? Med Hypotheses (2006) 67(4):782–5. 10.1016/j.mehy.2006.04.029 16828235

[31] Masala S Paccagnini D Cossu D Brezar V Pacifico A Ahmed N Antibodies recognizing *Mycobacterium avium* paratuberculosis epitopes cross-react with the beta-cell antigen ZnT8 in Sardinian type 1 diabetic patients. PLoS One (2011) 6(10):e26931. 10.1371/journal.pone.0026931 22046415 PMC3203182

[32] Sechi LA Paccagnini D Salza S Pacifico A Ahmed N Zanetti S *Mycobacterium avium* subspecies paratuberculosis bacteremia in type 1 diabetes mellitus: an infectious trigger? Clin Infect Dis (2008) 46(1):148–9 10.1086/524084 18171233

[33] D’Amore M Lisi S Sisto M Cucci L Dow CT Molecular identification of *Mycobacterium avium* subspecies paratuberculosis in an Italian patient with Hashimoto’s thyroiditis and Melkersson-Rosenthal syndrome. J Med Microbiol (2010) 59(Pt 1):137–9 10.1099/jmm.0.013474-0 19797462

[34] Sisto M Cucci L D’Amore M Dow TC Mitolo V Lisi S. Proposing a relationship between *Mycobacterium avium* subspecies paratuberculosis infection and Hashimoto’s thyroiditis. Scand J Infect Dis (2010) 42(10):787–90. 10.3109/00365541003762306 20429717

[35] Masala S Cossu D Palermo M Sechi LA. Recognition of zinc transporter 8 and MAP3865c homologous epitopes by Hashimoto’s thyroiditis subjects from Sardinia: a common target with type 1 diabetes? PLoS One (2014) 9(5):e97621. 10.1371/journal.pone.0097621 24830306 PMC4022723

[36] Pinna A Masala S Blasetti F Maiore I Cossu D Paccagnini D Detection of serum antibodies cross-reacting with *Mycobacterium avium* subspecies paratuberculosis and beta-cell antigen zinc transporter 8 homologous peptides in patients with high-risk proliferative diabetic retinopathy. PLoS One (2014) 9(9):e107802. 10.1371/journal.pone.0107802 25226393 PMC4166466

[37] Cossu D Cocco E Paccagnini D Masala S Ahmed N Frau J Association of *Mycobacterium avium* subsp. paratuberculosis with multiple sclerosis in Sardinian patients. PLoS One (2011) 6(4):e18482 10.1371/journal.pone.0018482 21533236 PMC3076380

[38] Cossu D Mameli G Masala S Cocco E Frau J Marrosu MG Evaluation of the humoral response against mycobacterial peptides, homologous to MOG35-55, in multiple sclerosis patients. J Neurol Sci (2014) 347(1–2):78–81. 10.1016/j.jns.2014.09.023 25271190

[39] Cossu D Masala S Cocco E Paccagnini D Tranquilli S Frau J Association of *Mycobacterium avium* subsp. paratuberculosis and SLC11A1 polymorphisms in Sardinian multiple sclerosis patients. J Infect Dev Ctries (2013) 7(3):203–7. 10.3855/jidc.2737 23492997

[40] Gazouli M Sechi L Paccagnini D Sotgiu S Arru G Nasioulas G NRAMP1 polymorphism and viral factors in Sardinian multiple sclerosis patients. Can J Neurol Sci (2008) 35(4):491–4. 10.1017/S0317167100009173 18973068

[41] Ates O Kurt S Bozkurt N Karaer H. NRAMP1 (SLC11A1) variants: genetic susceptibility to multiple sclerosis. J Clin Immunol (2010) 30(4):583–6. 10.1007/s10875-010-9422-5 20405176

[42] Cossu D Masala S Cocco E Paccagnini D Frau J Marrosu MG Are *Mycobacterium avium* subsp. paratuberculosis and Epstein-Barr virus triggers of multiple sclerosis in Sardinia? Mult Scler (2012) 18(8):1181–4. 10.1177/1352458511433430 22261119

[43] Mameli G Cossu D Cocco E Masala S Frau J Marrosu MG Epstein-Barr virus and *Mycobacterium avium* subsp. paratuberculosis peptides are cross recognized by anti-myelin basic protein antibodies in multiple sclerosis patients. J Neuroimmunol (2014) 270(1–2):51–5 10.1016/j.jneuroim.2014.02.013 24642384

[44] Mameli G Cossu D Cocco E Masala S Frau J Marrosu MG EBNA-1 IgG titers in Sardinian multiple sclerosis patients and controls. J Neuroimmunol (2013) 264(1–2):120–2. 10.1016/j.jneuroim.2013.07.017 24099984

[45] Cossu D Masala S Frau J Mameli G Marrosu MG Cocco E Antigenic epitopes of MAP2694 homologous to T-cell receptor gamma-chain are highly recognized in multiple sclerosis Sardinian patients. Mol Immunol (2014) 57(2):138–40. 10.1016/j.molimm.2013.09.001 24091296

[46] Cossu D Masala S Sechi LA A Sardinian map for multiple sclerosis. Future Microbiol (2013) 8(2):223–32 10.2217/fmb.12.135 23374127

[47] Soares RM Dias AT De Castro SB Alves CC Evangelista MG Da Silva LC Optical neuritis induced by different concentrations of myelin oligodendrocyte glycoprotein presents different profiles of the inflammatory process. Autoimmunity (2013) 46(7):480–5. 10.3109/08916934.2013.796938 24083391

[48] Cossu D Mameli G Galleri G Cocco E Masala S Frau J Human interferon regulatory factor 5 homologous epitopes of Epstein-Barr virus and *Mycobacterium avium* subsp. paratuberculosis induce a specific humoral and cellular immune response in multiple sclerosis patients. Mult Scler (2014) 12. 25392335 10.1177/1352458514557304

[49] Frau J Cossu D Coghe G Lorefice L Fenu G Porcu G Role of interferon-beta in *Mycobacterium avium* subspecies paratuberculosis antibody response in Sardinian MS patients. J Neurol Sci (2015) 349(1–2):249–50. 10.1016/j.jns.2015.01.004 25598492

[50] Blau B. Familial granulomatous arthritis, iritis, and rash. J Pediatr (1985) 107(5):689–93. 10.1016/S0022-3476(85)80394-2 4056967

[51] Hampe J Grebe J Nikolaus S Solberg C Croucher PJ Mascheretti S Association of NOD2 (CARD 15) genotype with clinical course of Crohn’s disease: a cohort study. Lancet (2002) 359(9318):1661–5. 10.1016/S0140-6736(02)08590-2 12020527

[52] Inohara N Ogura Y Fontalba A Gutierrez O Pons F Crespo J Host recognition of bacterial muramyl dipeptide mediated through NOD2: implications for Crohn’s disease. J Biol Chem (2003) 278(8):5509–12. 10.1074/jbc.C200673200 12514169

[53] Hansen JM Golchin SA Veyrier FJ Domenech P Boneca IG Azad AK N-glycolylated peptidoglycan contributes to the immunogenicity but not pathogenicity of *Mycobacterium tuberculosis*. J Infect Dis (2014) 209(7):1045–54. 10.1093/infdis/jit622 24265438

[54] Kanazawa N Okafuji I Kambe N Nishikomori R Nakata-Hizume M Nagai S Early-onset sarcoidosis and CARD15 mutations with constitutive nuclear factor-κB activation: common genetic etiology with Blau syndrome. Blood (2005) 105(3):1195–7. 10.1182/blood-2004-07-2972 15459013

[55] Miceli-Richard C Lesage S Rybojad M Prieur AM Manouvrier-Hanu S Häfner R CARD15 mutations in Blau syndrome. Nat Genet (2001) 29(1):19–20 10.1038/ng720 11528384

[56] Wang X Kuivaniemi H Bonavita G Mutkus L Mau U Blau E CARD15 mutations in familial granulomatosis syndromes: a study of the original Blau syndrome kindred and other families with large-vessel arteritis and cranial neuropathy. Arthritis Rheum (2002) 46(11):3041–5. 10.1002/art.10618 12428248

[57] Hugot J-P Chamaillard M Zouali H Lesage S Cézard JP Belaiche J Association of NOD2 leucine-rich repeat variants with susceptibility to Crohn’s disease. Nature (2001) 411(6837):599–603. 10.1038/35079107 11385576

[58] Lesage S Zouali H Cézard JP Colombel JF Belaiche J Almer S CARD15/NOD2 mutational analysis and genotype-phenotype correlation in 612 patients with inflammatory bowel disease. Am J Hum Genet (2002) 70(4):845–57. 10.1086/339432 11875755 PMC379113

[59] Sechi LA Gazouli M Ikonomopoulos J Lukas JC Scanu AM Ahmed N *Mycobacterium avium* subsp. paratuberculosis, genetic susceptibility to Crohn’s disease, and Sardinians: the way ahead. J Clin Microbiol (2005) 43(10):5275–7. 10.1128/JCM.43.10.5275-5277.2005 16207995 PMC1248481

[60] Rose CD Doyle TM McIlvain-Simpson G Coffman JE Rosenbaum JT Davey MP Blau syndrome mutation of CARD15/NOD2 in sporadic early onset granulomatous arthritis. J Rheumatol (2005) 32(2):373–5. 15693102

[61] Wouters CH Maes A Foley KP Bertin J Rose CD. Blau syndrome, the prototypic auto-inflammatory granulomatous disease. Pediatr Rheumatol Online J (2014) 12:33. 10.1186/1546-0096-12-33 25136265 PMC4136643

[62] Lacher M Helmbrecht J Schroepf S Koletzko S Ballauff A Classen M NOD2 mutations predict the risk for surgery in pediatric-onset Crohn’s disease. J Pediatr Surg (2010) 45(8):1591–7. 10.1016/j.jpedsurg.2009.10.046 20713205

[63] Franke A McGovern DP Barrett JC Wang K Radford-Smith GL Ahmad T Genome-wide meta-analysis increases to 71 the number of confirmed Crohn’s disease susceptibility loci. Nat Genet (2010) 42(12):1118–25. 10.1038/ng.717 21102463 PMC3299551

[64] Sechi LA Gazouli M Sieswerda LE Molicotti P Ahmed N Ikonomopoulos J Relationship between Crohn’s disease, infection with *Mycobacterium avium* subspecies paratuberculosis and SLC11A1 gene polymorphisms in Sardinian patients. World J Gastroenterol (2006) 12(44):7161–4. 17131479 10.3748/wjg.v12.i44.7161 PMC4087778

[65] Canonne-Hergaux F Gruenheid S Govoni G Gros P. (1999) The Nramp1 protein and its role in resistance to infection and macrophage function. Proc Assoc Am Physicians (1999) 111:283–9. 10.1046/j.1525-1381.1999.99236.x 10417735

[66] Lapham AS Phillips ES Barton CH. Transcriptional control of Nramp1: a paradigm for the repressive action of c-Myc. Biochem Soc Trans (2004) 32(Pt 6):1084–6. 10.1042/BST0321084 15506972

[67] Wyllie S Seu P Goss JA. The natural resistance-associatedmacrophage protein 1 Slc11a1 (formerly Nramp1) and iron metabolism in macrophages. Microbes Infect (2002) 4(3):351–9. 10.1016/S1286-4579(02)01548-4 11909746

[68] Hackam DJ Rotstein OD Zhang W Gruenheid S Gros P Grinstein S. Host resistance to intracellular infection: mutation of natural resistance-associated macrophage protein 1 (Nramp1) impairs phagosomal acidification. J Exp Med (1998) 188(2):351–64. 10.1084/jem.188.2.351 9670047 PMC2212455

[69] Dubaniewicz A Jamieson SE Dubaniewicz-Wybieralska M Fakiola M Nancy Miller E Blackwell JM. Association between SLC11A1 (formerly NRAMP1) and the risk of sarcoidosis in Poland. Eur J Hum Genet (2005) 13(7):829–34. 10.1038/sj.ejhg.5201370 15702130

[70] Stienstra Y van der Werf TS Oosterom E Nolte IM van der Graaf WT Etuaful S Susceptibility to Buruli ulcer is associated with the SLC11A1 (NRAMP1) D543N polymorphism. Genes Immun (2006) 7(3):185–9. 10.1038/sj.gene.6364281 16395392

[71] Ruiz-Larrañaga O Garrido JM Manzano C Iriondo M Molina E Gil A Identification of single nucleotide polymorphisms in the bovine solute carrier family 11 member 1 (SLC11A1) gene and their association with infection by *Mycobacterium avium* subspecies paratuberculosis. J Dairy Sci (2010) 93(4):1713–21. 10.3168/jds.2009-2438 20338449

[72] Korou LM Liandris E Gazouli M Ikonomopoulos J. Investigation of the association of the SLC11A1 gene with resistance/sensitivity of goats (*Capra hircus*) to paratuberculosis. Vet Microbiol (2010) 144(3–4):353–8. 10.1016/j.vetmic.2010.01.009 20188496

[73] Purdie AC Plain KM Begg DJ de Silva K Whittington RJ. Candidate gene and genome-wide association studies of *Mycobacterium avium* subsp. paratuberculosis infection in cattle and sheep: a review. Comp Immunol Microbiol Infect Dis (2011) 34(3):197–208. 10.1016/j.cimid.2010.12.003 21216466

[74] Roupie V Rosseels V Piersoel V Zinniel DK Barletta RG Huygen K. Genetic resistance of mice to *Mycobacterium paratuberculosis* is influenced by Slc11a1 at the early but not at the late stage of infection. Infect Immun (2008) 76(5):2099–105. 10.1128/IAI.01137-07 18285491 PMC2346681

[75] Blackwell JM Searle S Mohamed H White JK. Divalent cation transport and susceptibility to infectious and autoimmune disease: continuation of the Ity/Lsh/Bcg/Nramp1/Slc11a1 gene story. Immunol Lett (2003) 85(2):197–203. 10.1016/S0165-2478(02)00231-6 12527228

[76] Hatta M Ratnawati Tanaka M Ito J Shirakawa T Kawabata M. NRAMP1/SLC11A1 gene polymorphisms and host susceptibility to *Mycobacterium tuberculosis* and *M. leprae* in South Sulawesi, Indonesia. Southeast Asian J Trop Med Public Health (2010) 41(2):386–94. 20578522

[77] Bellamy R Ruwende C Corrah T McAdam KP Whittle HC Hill AV. Variations in the NRAMP1 gene and susceptibility to tuberculosis in West Africans. N Engl J Med (1998) 338:640–4. 10.1056/NEJM199803053381002 9486992

[78] Ates O Dalyan L Musellim B Hatemi G Turker H Ongen G NRAMP1 (SLC11A1) gene polymorphisms that correlate with autoimmune versus infectious disease susceptibility in tuberculosis and rheumatoid arthritis. Int J Immunogenet (2009) 36:15–9. 10.1111/j.1744-313X.2008.00814.x 19055603

[79] Kotze MJ de Villiers JN Rooney RN Grobbelaar JJ Mansvelt EP Bouwens CS Analysis of the NRAMP1 gene implicated in iron transport: association with multiple sclerosis and age effects. Blood Cells Mol Dis (2001) 27:44–53. 10.1006/bcmd.2000.0349 11358358

[80] Gazouli M Atsaves V Mantzaris G Economou M Nasioulas G Evangelou K Role of functional polymorphisms of NRAMP1 gene for the development of Crohn’s disease. Inflamm Bowel Dis (2008) 14:1323–30. 10.1002/ibd.20488 18454481

[81] Kotlowski R Bernstein CN Silverberg MS Krause DO. Population-based case-control study of alpha 1-antitrypsin and SLC11A1 in Crohn’s disease and ulcerative colitis. Inflamm Bowel Dis (2008) 14:1112–7. 10.1002/ibd.20425 18340647

[82] Paccagnini D Sieswerda L Rosu V Masala S Pacifico A Gazouli M Linking chronic infection and autoimmune diseases: *Mycobacterium avium* subspecies par atuberculosis, SLC11A1 polymorphisms and type-1 diabetes mellitus. PLoS One (2009) 214(9):e7109. 10.1371/journal.pone.0007109 19768110 PMC2740822

[83] Masala S Cossu D Pacifico A Molicotti P Sechi LA. Sardinian type 1 diabetes patients, transthyretin and *Mycobacterium avium* subspecies paratuberculosis infection. Gut Pathog (2012) 4(1):24. 10.1186/1757-4749-4-24 23270597 PMC3543228

[84] Takahashi K Satoh J Kojima Y Negoro K Hirai M Hinokio Y Promoter polymorphism of SLC11A1 (formerly NRAMP1) confers susceptibility to autoimmune type 1 diabetes mellitus in Japanese. Tissue Antigens (2004) 63(3):231–6. 10.1111/j.1399-0039.2004.000172.x 14989712

[85] Oldstone MB Molecular mimicry and autoimmune disease. Cell (1987) 50(6):819–20 10.1016/0092-8674(87)90507-1 3621346

[86] Raska M Weigl E. Heat shock proteins in autoimmune diseases. Biomed Pap Med Fac Univ Palacky Olomouc Czech Repub (2005) 149(2):243–9. 10.5507/bp.2005.033 16601763

[87] Guilherme L Faé K Oshiro SE Kalil J Molecular pathogenesis of rheumatic fever and rheumatic heart disease. Expert Rev Mol Med (2005) 7(28):1–15 10.1017/S146239940501015X 16336741

[88] Kaplan MH SVEC KH. Immunologic relation of streptococcal and tissue antigens. III. Presence in human sera of streptococcal antibody cross-reactive with heart tissue. Association with streptococcal infection, rheumatic fever, and glomerulonephritis. J Exp Med (1964) 119:65166. 10.1084/jem.119.4.651 14151105 PMC2137853

[89] Eisenbarth GS Type I diabetes mellitus. A chronic autoimmune disease. N Engl J Med (1986) 314(21):1360–8 10.1056/NEJM198605223142106 3517648

[90] Davis W Madsen-Bouterse S. Crohn’s disease and *Mycobacterium avium* subsp. paratuberculosis: the need for a study is long overdue. Vet Immunol Immunopathol (2012) 145(1–2):1–6. 10.1016/j.vetimm.2011.12.005 22209202 PMC3273645

[91] Masala S Zedda MA Cossu D Ripoli C Palermo M Sechi LA. Zinc transporter 8 and MAP3865c homologous epitopes are recognized at T1D onset in Sardinian children. PLoS One (2013) 8(5):e63371. 10.1371/journal.pone.0063371 23696819 PMC3656963

[92] Masala S Cossu D Piccinini S Rapini N Massimi A Porzio O Recognition of zinc transporter 8 and MAP3865c homologous epitopes by new-onset type 1 diabetes children from continental Italy. Acta Diabetol (2014) 51(4):577–85. 10.1007/s00592-014-0558-2 24496951

[93] Masala S Cossu D Piccinini S Rapini N Mameli G Manca Bitti ML Proinsulin and MAP3865c homologous epitopes are a target of antibody response in new-onset type 1 diabetes children from continental Italy. Pediatr Diabetes J (2015). (in press). 10.1111/pedi.12269 25720593

[94] Rani PS Sechi LA Ahmed N. *Mycobacterium avium* subsp. paratuberculosis as a trigger of type-1 diabetes: destination Sardinia, or beyond? Gut Pathog (2010) 2(1):1. 10.1186/1757-4749-2-1 20350307 PMC2867798

[95] Scotto M Afonso G Larger E Raverdy C Lemonnier FA Carel JC Zinc transporter (ZnT)8(186-194) is an immunodominant CD8+ T cell epitope in HLA-A2+ type 1 diabetic patients. Diabetologia (2012) 55(7):2026–31. 10.1007/s00125-012-2543-z 22526607 PMC3740540

[96] Cossu A Ferrannini E Fallahi P Antonelli A Sechi LA. Antibodies recognizing specific *Mycobacterium avium* subsp. paratuberculosis’s MAP3738c protein in type 1 diabetes mellitus children are associated with serum Th1 (CXCL10) chemokine. Cytokine (2013) 61(2):337–9. 10.1016/j.cyto.2012.11.008 23265968

[97] Kawasaki E Type 1 diabetes and autoimmunity. Clin Pediatr Endocrinol (2014) 23(4):99–105 10.1297/cpe.23.99 25374439 PMC4219937

[98] Rogowicz-Frontczak A Zozuliłska-Ziołkiewicz D Litwinowicz M Niedzwiecki P Wyka K Wierusz-Wysocka B. Are zinc transporter type 8 antibodies a marker of autoimmune thyroiditis in non-obese adults with new-onset diabetes? Eur J Endocrinol (2014);170(4):651–8. 10.1530/EJE-13-0901 24480135

[99] Parsell DA Lindquist S The function of heat shock proteins in stress tolerance: degradation and reactivation of damaged proteins. Annu Rev Genet (1993) 27:437–96 10.1146/annurev.ge.27.120193.002253 8122909

[100] Lamb JR Young DB. T cell recognition of stress proteins. A link between infectious and autoimmune disease. Mol Biol Med (1990) 7(4):311–21. 1978220

[101] Jarjour WN Jeffries BD Davis JS IV Welch WJ Mimura T Winfield JB. Autoantibodies to human stress proteins. A survey of various rheumatic and other inflammatory diseases. Arthritis Rheum (1991) 34(9):1133–8. 10.1002/art.1780340909 1930332

[102] Moudgil KD Chang TT Eradat H Chen AM Gupta RS Brahn E Diversification of T cell responses to carboxy-terminal determinants within the 65 kD heat-shock protein is involved in regulation of autoimmune arthritis. J Exp Med (1997) 185(7):1307–16. 10.1084/jem.185.7.1307 9104817 PMC2196249

[103] Cossu D Masala S Frau J Cocco E Marrosu MG Sechi LA. Anti *Mycobacterium avium* subsp. paratuberculosis heat shock protein 70 antibodies in the sera of Sardinian patients with multiple sclerosis. J Neurol Sci (2013) 335(1–2):131–3. 10.1016/j.jns.2013.09.011 24075312

[104] Quayle AJ Wilson KB Li SG Kjeldsen-Kragh J Oftung F Shinnick T Peptide recognition, T cell receptor usage and HLA restriction elements of human heat-shock protein (hsp) 60 and mycobacterial 65 kDa hsp-reactive T cell clones from rheumatoid synovial fluid. Eur J Immunol (1992) 22(5):1315–22. 10.1002/eji.1830220529 1577070

[105] Miyata M Kogure A Sato H Kodama E Watanabe H Ohira H Detection of antibodies to 65 KD heat shock protein and to human superoxide dismutase in autoimmune hepatitis-molecular mimicry between 65 KD heat shock protein and superoxide dismutase. Clin Rheumatol (1995) 14(6):673–7. 10.1007/BF02207935 8608687

[106] Vilagut L Pares A Vinas O Vila J Jiménez de Anta MT Rodés J. Antibodies to mycobacterial 65 kD heat shock protein cross-react with the main mitochondrial antigens in patients with primary biliary cirrhosis. Eur J Clin Invest (1997) 27(8):667–72. 10.1046/j.1365-2362.1997.1690724.x 9279530

[107] Danieli MG Candela M Ricciatti AM Reginelli R Danieli G Cohen IR Antibodies to mycobacterial 65 kDa heat shock protein in systemic sclerosis (scleroderma). J Autoimmun (1992) 5(4):443–5. 10.1016/0896-8411(92)90004-A 1418288

[108] Yokota S Tsubaki S Kuriyama T Shimizu H Ibe M Mitsuda T Presence in Kawasaki disease of antibodies to mycobacterial heatshock protein HSP65 and autoantibodies to epitopes of human HSP65 cognate antigen. Clin Immunol Immunopathol (1993) 67:163–70. 10.1006/clin.1993.1060 7686092

[109] Direskeneli H Saruhan-Direskeneli G. The role of heat shock proteins in Behcet’s disease. Clin Exp Rheumatol (2003) 21(Suppl 30):S44–8. 14727460

[110] Aggarwal A Chag M Sinha N Naik S. Takayasu’s arteritis: role of *Mycobacterium tuberculosis* and its 65 kDa heat shock protein. Int J Cardiol (1996) 55(1):49–55. 10.1016/0167-5273(96)02660-5 8839810

[111] Hermon-Taylor J Bull T. Crohn’s disease caused by *Mycobacterium avium* subspecies paratuberculosis: a public health tragedy whose resolution is long overdue. J Med Microbiol (2002) 51(1):3–6. 11800469 10.1099/0022-1317-51-1-3

[112] Hermon-Taylor J Treatment with drugs active against *Mycobacterium avium* subspecies paratuberculosis can heal Crohn’s disease: more evidence for a neglected public health tragedy. Dig Liver Dis (2002) 34(1):9–12 10.1016/S1590-8658(02)80052-4 11926580

[113] De Buck J Shaykhutdinov R Barkema HW Vogel HJ. Metabolomic profiling in cattle experimentally infected with *Mycobacterium avium* subsp. paratuberculosis. PLoS One (2014) 9(11):e111872. 10.1371/journal.pone.0111872 25372282 PMC4221196

[114] Gitlin L Borody TJ Chamberlin W Campbell J. *Mycobacterium avium* ss paratuberculosis-associated diseases: piecing the Crohn’s puzzle together. J Clin Gastroenterol (2012) 46(8):649–55. 10.1097/MCG.0b013e31825f2bce 22858515

[115] Feller M Huwiler K Schoepfer A Shang A Furrer H Egger M. Long-term antibiotic treatment for Crohn’s disease: systematic review and meta-analysis of placebo-controlled trials. Clin Infect Dis (2010) 50(4):473–80. 10.1086/649923 20067425

[116] Available from: http://clinicaltrials.gov/ct2/show/NCT01951326?term=rhb-104&rank=2 last viewed 11.12.14

[117] Available from: http://clinicaltrials.gov/ct2/show/NCT01717664?term=rhb-104&rank=1 last viewed 11.12.14

